# Seasonal Variations in Semen Quality, Testosterone Levels, and Scrotal Size following Dietary Flaxseed Oil and Ascorbic Acid in South African Indigenous Rams

**DOI:** 10.3390/ani13071213

**Published:** 2023-03-30

**Authors:** Jabulani Nkululeko Ngcobo, Tshimangadzo Lucky Nedambale, Takalani Judas Mpofu, Khathutshelo Agree Nephawe, Tlou Caswell Chokoe, Fhulufhelo Vincent Ramukhithi

**Affiliations:** 1 Department of Animal Sciences, Tshwane University of Technology, Private Bag X680, Pretoria 0001, South Africa; nedambaletl@tut.ac.za (T.L.N.); mpofutj@tut.ac.za (T.J.M.); nephaweka@tut.ac.za (K.A.N.); 2 Agricultural Research Council, Germplasm, Conservation, Reproductive Biotechnologies, Private Bag X02, Irene 0062, South Africa; ramukhithif@arc.agric.za; 3 Department of Agriculture, Land Reform and Rural Development, Directorate Farm Animal Genetic Resource, Private Bag X250, Pretoria 0001, South Africa; tlouc@dalrrd.gov.za

**Keywords:** conservation, omega n-3, semen quality, docosahexaenoic acid, fertility

## Abstract

**Simple Summary:**

The impact of climate change around the world cannot be overstated. South African indigenous sheep face the threat of extinction. This study was aimed at evaluating the seasonal variations in semen quality, testosterone levels, and scrotal size following dietary flaxseed oil and ascorbic acid in South African indigenous rams. Total motility did not differ significantly (*p* > 0.05) between the seasons when negative and (PC) were supplemented; nevertheless, there was an improvement when FLAX, ASCA, and FLAX + ASCA were supplemented. Testosterone levels were significantly influenced by the seasons when negative and PC diets were supplemented. It was observed that flaxseed oil and ascorbic acid can reverse the seasonal variations in semen quality and testosterone levels.

**Abstract:**

The purpose of this study was to determine the seasonal variations in semen quality, testosterone levels, and scrotal size, following dietary flaxseed oil and ascorbic acid in South African indigenous rams. A total of 22 South African indigenous rams were randomly distributed into five treatment diets from June 2021 to May 2022 (12 months). To allow for the spermatogenesis period, semen was collected after sixty days of dietary supplementation with treatment diets. Blood was collected twice a week using an 18-gauge needle and vacutainer tubes and sent to the laboratory for testosterone analysis. Semen and blood collection were repeated eight times each season. The scrotal size (circumference, length, and width) was measured using a flexible measuring tape. Data was subjected to the General Linear Model (GLM) in Minitab^®^ 2017. Treatment means were separated using Fisher’s *t*-test and considered significantly different when the *p*-value was less than 0.05. Seasons and diet had an effect on progression, total motility, and testosterone levels. For instance, NC during the spring season had the lowest progressive motility (42.84 ± 5.32), followed by the summer (49.38 ± 4.49), winter (62.46 ± 4.35), and autumn (63.26 ± 3.58). Notably, when treatment diets were introduced, improvements were realized, and there were significant differences (*p* < 0.05) among the seasons following supplementation of FLAX, ASCA, and FLAX + ASCA, except for FLAX in the autumn season (53.83 ± 4.16). Total motility did not differ significantly (*p* > 0.05) between the seasons when the NC and PC diets were supplemented; nevertheless, there was an improvement when FLAX, ASCA, and FLAX + ASCA were supplemented. Testosterone levels were significantly influenced by the seasons when negative and PC diets were supplemented. It is noteworthy that supplementing FLAX + ASCA can reverse the influence of the season on the testosterone levels (spring, 27.52 ± 4.42; summer, 20.23 ± 5.11; autumn, 25.24 ± 3.96; and winter, 25.92 ± 4.42). In conclusion, seasons do affect semen quality and testosterone levels of South African indigenous rams. However, flaxseed oil and ascorbic acid can reverse the seasonal variations in semen quality and testosterone levels.

## 1. Introduction

In the year 2022, global hunger jumped to 9.8%, and the gender gap in food security rose; hence, about 3.1 billion people could not afford a healthy diet [1]. This led to the estimation that about 670 million people will face hunger by 2030 [1]. This happens at a time when climate change is a challenge in developing countries like South Africa [2]. Therefore, these factors will require a strategy in the agricultural sector to mitigate these challenges, including the improvement and conservation of indigenous genetic materials for future use or the development of adapted breeds for meat purposes. Mavule et al. [3] stated that South African indigenous sheep can walk longer distances in search of food and water and have a clear role in developing locally developed meat-type breeds [4].

South African indigenous sheep breeds (Zulu, BaPedi, and Namaqua Afrikaner) are facing the threat of extinction [5]. The South African government has set up conservation programs for these breeds [4]. However, conservation programs aiming to maintain genetic diversity with reproductive biotechnology require a basic understanding of reproductive biology and how it is influenced by environmental factors [6]. This is necessary because lambing in a season with low feed availability and a high disease load negatively affects flock performance. On the other hand, dispersed lambing leads to low selection intensity and thus poor genetic progress due to the ineffective selection of replacement ewes available in each season [7]. Again, lambing in an unfavorable season affects the flock’s productivity through the flock’s inability to survive and poor pre-weaning growth. For instance, lambs born in the post-rain and dry season periods survive better [8] than those born in the wet season due to internal parasites as one of the main factors [9].

The combination of genetic resource banks and advanced reproductive technologies offers an outstanding benefit in terms of maintaining biodiversity and assisting conservation programs for highly endangered breeds [10]. This is necessary for the indigenous breeds since they are being replaced by more productive breeds, thus resulting in a loss of genetic traits that would be needed in the future for cross-breed adaptation [11]. Nevertheless, enormous factors should be considered when constructing the sheep genetic resource banks because reproductive efficiency varies due to various factors, including breed [12], latitude [13], climate [14], season [15,16], nutrition [17], and circulating gonadotropin concentration [18]. Evaluating the semen samples for semen parameters such as semen volume, sperm concentration, sperm motility parameters, and morphology can be used to eliminate the use of infertile or sub-fertile males and trace seasonality in rams.

Fortunately, poor flock reproductive performance in ewes associated with the season can be mimicked using exogenous hormones to program lambing at a favorable season of the year, where pastures or veld are greener, in order to reduce mortality rates [19]. However, seasonal manifestation in rams is not well understood because some breeds show little or no seasonal effect [20,21,22]. Despite the seasonal effects, nutrition is another factor affecting semen production, spermatogenic tissues, testicular interstitial tissues, and the sexual behavior of rams [23]. Flaxseed oil contains 58% linolenic acid, an important antioxidant that benefits animal health [24]. Flaxseed oil also contains stearic, oleic, linoleic, and palmitic acids, all of which are high in vitamins, including vitamin E [25]. The role of flaxseed oil in improving semen quality and reproduction activities in poultry [26], turkeys [27], boars [28], and cattle [29,30] is well known. Based on our knowledge, however, there has been no study to date that has evaluated the influence of omega-3 fatty acids (from flaxseed oil) and ascorbic acid on semen quality and testosterone levels in different seasons of the year. As a result, this study aimed to evaluate the effects of the season after supplementing with flaxseed oil and ascorbic acid.

## 2. Materials and Methods

### 2.1. Study Ethics and Animals

All study procedures were approved by both the Agricultural Research Council Animal Ethics Committee (APAEC) with a reference number: APAEC 2019/23, and the Tshwane University of Technology Animal Research Ethics Committee (TUT-AREC) with a reference number: AREC 2020/05/001. The research was carried out at the Irene Animal Production Institute of the Agricultural Research Council, which specializes in reproductive biotechnologies, conservation, and germplasm (GCRB).

A total of twenty-two South African indigenous rams (8 BaPedi, 9 Zulu, and 5 Namaqua Afrikaner, with an average body weight of 64.4 ± 1.6 kg) were randomly distributed into five treatment diets from June 2021 to May 2022 (12 months), as described by Ngcobo et al. [31].

### 2.2. Measurement of Scrotal Dimensions

The scrotal circumference was measured using the flexible measuring tape, as described by Harighi et al. [32]. The scrotal length and scrotal width were measured using the Vernier caliper following a proper restraint of rams as suggested by Perumal et al. [33].

### 2.3. Experimental Design

The four seasons of the year are divided into four distinct periods: spring, summer, autumn, and winter. In South Africa, the spring season starts from September to November, summer from December to February, autumn from March to May, and winter starts from June to August [34]. In South Africa, the winter season has the shortest day length (10.5 h), followed by autumn (11.2 h), spring (12.6 h), and summer season having the longest (13.4 h) day length [34].

Four treatment diets (PC, FLAX, ASCA, and FLAX + ASCA) were formulated according to the National Research Council, 1987. The fifth diet (NC) was a standard feed diet in use currently at the Agricultural Research Council. Following feed formulation and mixing, feed samples were taken and stored in 500 g containers and labeled. These feed samples were taken to the South African National Accreditation System (SANAS) accredited laboratory for fatty acid profiling. The method number POL 015 was used for fatty acid profiling based on AOCS Ce2-66. Feed samples were collected after feed mixing and were taken to the SANAS-accredited laboratory for proximate analysis (Table 1). Formulated feed was supplemented for 60 days without semen collection to allow the proper influence of feed on spermatogenesis.

### 2.4. Semen Collection, Processing, and Evaluation

Semen was collected using an artificial vagina as described by Bopape et al. [35] from June 2021 to May 2022 (four seasons). Three weeks of training were provided for the rams to ensure smoothness and limit injuries during semen collection. Induced estrus ewes were neck clamped and used as libido stimuli [21]. Each ram was allocated at least 3 min to mount for successful semen collection. After semen collection, semen was transferred to a 15-mL Falcon tube and stored at 37 °C in a thermo-flask (Thermo Fisher Scientific, Randburg, South Africa). Semen and blood collection were repeated eight times each season. Semen was transported to the GCRB laboratory for evaluation within 30 min after collection.

Semen volumes were collected and placed into falcon tubes, and the volumes were recorded in mL [31]. Semen pH was evaluated using an Oakton pH meter (Eutech Instrument, Cyber-Scan pH 11/110, Singapore). The Oakton pH meter was rinsed with clean drinking water and calibrated with pH buffers (Eutech Instrument, Cyber-Scan pH 11/110, Singapore) of 4, 7, and 10 before being used to measure pH levels [31].

Sperm concentration was measured using a spectrophotometer [36] at 534 nm absorbance. Sodium citrate (1.45 g) was dissolved in a 50 mL tube and used to measure the sperm concentration. A 7.5 g sample of semen was mixed with 1500 µL of prepared sodium citrate.

Using the Computer-Aided Sperm Analyzer (CASA) and Sperm Class Analyzer (SCA) 5.0 version, fresh semen samples were assessed for sperm motility parameters (total, progressive, non-progressive, rapid, medium, and slow motility). In the Eppendorf tube, five microliters of semen and one hundred microliters of swim-up medium (a tris-based extender free of egg yolk and glycerol) were combined to assess motility. Following a semen and swim-up medium (SWM) mixture, five µL of the mixture was pipetted and placed on a warmed microscope slide (76 × 26 × 1 mm, Sigma-Aldrich Chemie GmbH, Steinheim am Albuch, Germany) before being gently enclosed with a coverslip (22 × 22 mm, Germany) [31]. The enclosed slide was thereafter placed over a warm CASA stage (Omron) adjusted to 37 °C, under the CASA camera microscope at a magnification of 10× Ph1 BM. For each sample, three fields were randomly selected, containing 150 to 200 spermatozoa that were captured and examined.

The eosin-nigrosin stain was used to stain semen for sperm morphology and viability at a ratio of 5:20 (semen: eosin-nigrosin). To avoid harming sperm cells, the mixture was pipetted onto the microscope slide and stained using a second microscope slide held at a 45-degree angle. For sperm viability, two hundred sperm cells were counted and converted to 100%. For sperm morphology, two hundred sperm cells were also counted and subdivided into normal and abnormal sperm cells. Primary abnormalities (knobbed acrosome, large/swollen head, small head, misshapen head, double tail, double head, and abaxial mid-piece), secondary abnormalities (proximal droplets, bent tail, distal droplets, and loose-head), and tertiary abnormalities (reacted acrosome, coiled tail, loose tail, and terminal droplets) were identified in sperm cells.

With a few changes, Maxwell and Johnson’s Hyper-Osmotic Swelling Test (HOST) was used to assess membrane permeability, as further described by Ngcobo et al. [31]. Following the mixing of the semen and the HOST, the solution was then incubated for 30 min at 37 °C in a 5% CO_2_ incubator (MCO-20 AIC Sanyo^®^ CO_2_ incubator (Sanyo, Japan)) [37]. Thereafter, samples were smeared on the microscope slide (76 × 26 × 1 mm, Germany) gently at a 45° angle, and air-dried. Slides were examined under a 400× phase-contrast microscope without exposure to ambient light. Sperm cells with coiled tails were observed to have good membrane integrity under the microscope, while those with straight tails were observed to be damaged sperm cells. On each slide, 300 spermatozoa were counted in total.

For malondialdehyde levels, semen concentration was initially determined, and then samples were standardized to 5.29 × 109 ± 0.36 [31] using a non-egg-yolk and glycerol tris-based extender [38]. Thereafter, the standardized samples were mixed with 1 mL of trichloroacetic acid (TCA) to precipitate proteins [37]. The mixture was then centrifuged for 10 min at 1500× *g* force, according to [37]. After centrifugation, the sample solution was collected, and the supernatant was mixed with 0.67% TBA in a 1:1 ratio before boiling at 100 °C for 10 min and cooling to room temperature before analysis. The absorbance was measured with a spectrophotometer programmed to 534 nm.

The blood samples were collected on a weekly basis in different seasons. Collected blood was then kept at room temperature for ±5 h to allow serum separation. Thereafter, blood serum was kept at −20 °C in the fridge until analysis. The blood samples were then sent to the laboratory for analysis using the ELISA kits (Demeditec, Diagnostics GmbH, D-24145, Kiel, Germany) as per the manufacturer’s guidelines.

### 2.5. Statistical Analysis

To evaluate the possible seasonal variations on semen quality, testosterone levels, and scrotal size in different seasons of the year, a General Linear Model (GLM) was applied using Minitab 17^®^. Treatment means were separated using Fisher’s *t*-test and considered significantly different when the *p*-value was less than 0.05.

## 3. Results

This study evaluated seasonal variations in semen quality in South African indigenous rams. The season had a significant (*p* < 0.05) effect on the semen parameters. For instance, semen collected during spring and autumn had significantly (*p* < 0.05) higher sperm concentrations (0.93 and 0.84, respectively) in comparison to that collected during the autumn (0.71) and winter (0.70) seasons (Table 2). There was no significant difference (*p* > 0.05) in semen volume during the spring (0.88 mL), summer (0.91 mL), autumn (0.92 mL), and winter (0.92 mL) seasons. Semen pH was significantly higher (*p* < 0.05) during the spring (6.44 ± 0.11) and summer (6.32) seasons in comparison to that of the autumn (6.07) and winter (6.10) seasons.

The effects of seasons and treatment diets on semen quality were evaluated. Although there was a noticeable seasonal influence on semen quality and quantity (Table 3), the dietary inclusion of flaxseed oil and ascorbic acid played a significant role in reducing the seasonal effect. For instance, dietary inclusion of FLAX + ASCA during the spring season led to a significant (*p* < 0.05) increase in semen volume (1.20 ± 0.11) when compared to the NC (0.55 ± 0.11) and the PC (0.72 ± 0.11). A similar trend was observed in other seasons, where dietary inclusion of FLAX + ASCA improved the semen volume, although it could not differ from that of FLAX and ASCA.

Semen pH could not differ significantly (*p* > 0.05) in the NC, PC, and FLAX groups regardless of the season. The ASCA led to higher semen pH during the spring (7.00 ± 0.23) season when compared to the autumn (6.11 ± 0.16) but could not differ from that of the summer (6.44 ± 0.20) and winter (6.17 ± 0.19) seasons. The FLAX + ASCA, on the other hand, recorded a non-significant (*p* > 0.05) difference in semen pH among seasons.

Sperm concentration was affected by the season in the NC-fed rams, with the summer season recording the highest sperm concentration (0.94 ± 0.11) when compared to autumn (0.65 ± 0.09) and winter (0.53 ± 0.11) scoring the lowest. The PC, FLAX, ASCA, and FLAX + ASCA fed groups had a non-significant difference (*p* > 0.05) in sperm concentration in all seasons. Sperm concentrations across the table differed significantly only during the spring season, when the NC-treated group had the lowest sperm concentration.

The season had a significant (*p* < 0.05) effect on the sperm motility parameters. Although there was a non-significant (*p* > 0.05) different effect of season, progressive motility, non-progressive motility, total motility, and static differed with the season. The autumn season scored the lowest NPM (25.28 ± 1.62) but could not differ from that of the winter (28.79 ± 1.85) season (Table 4). The summer season had a higher TM (94.25 ± 1.12) when compared to the spring (91.01 ± 1.29) season; however, it could not differ from that observed in the autumn (92.16 ± 0.94) and winter (92.81 ± 1.07) seasons. The SM was significantly (*p* < 0.05) higher during the spring (8.68 ± 1.07) and lowest during the summer (5.48 ± 0.94) season.

The sperm speeds measured by CASA were also determined to be affected by the season; the corresponding results are presented in Table 4. The RM sperm was significantly (*p* < 0.05) higher during the autumn (60.78 ± 2.45) and winter (55.50 ± 2.81) seasons when compared to the spring (51.06 ± 3.37) and the summer (50.31 ± 2.94) seasons. The MM sperm was higher during the summer (23.11 ± 1.98), compared to the spring (16.69 ± 2.26), autumn (11.01 ± 1.65), and winter (16.63 ± 1.89) seasons. In terms of SM, there was a non-significant (*p* > 0.05) difference observed among the seasons.

The effect of seasons and treatment diets on sperm cell motility parameters was evaluated. Although seasons had a noticeable effect on the semen quality (see previous tables), once treatment diets were introduced, improvements were realized. For instance, progressive motility was the lowest in the NC (42.84 ± 5.32) in comparison to other dietary groups during the spring season (Table 5). Nevertheless, there was no significant difference in progressive motility during the spring season for the PC, FLAX, ASCA, and FLAX + ASCA. There was a noticeable improvement in progressive motility during the summer season, especially following the use of an ASCA (73.66 ± 4.60) as compared to that of the NC (49.38 ± 4.49). However, it did not differ from that of the PC (60.15 ± 4.36), FLAX (62.24 ± 4.28), or FLAX + ASCA (68.49 ± 4.28). Progressive motility during the autumn and winter seasons could not differ significantly (*p* > 0.05) between all treatment diets.

The NC yielded a significantly higher (*p* < 0.05) non-progressive motility than the PC (29.40 ± 4.42), FLAX (28.99 ± 4.36), ASCA (23.42 ± 4.60), and FLAX + ASCA (24.53 ± 4.36) during the spring season. Nevertheless, non-progressive motility could not differ significantly (*p* > 0.05) during the spring season for the PC, FLAX, ASCA, and FLAX + ASCA. Moreover, non-progressive motility did not differ significantly (*p* > 0.05) for all treatment diets during the autumn and winter seasons.

Total motility was higher in the PC (91.56 ± 2.56), FLAX (91.99 ± 2.53), and FLAX + ASCA (94.76 ± 2.53) than that of the NC (88.93 ± 2.62) and ASCA (87.79 ± 2.66) during the spring season. Noteworthy, there was no significant difference (*p* > 0.05) between the PC, FLAX, and FLAX + ASCA as well as between the NC and AA during the spring season. The NC group had the lowest (88.43 ± 2.21; total motility) during the summer season. There was no significant difference observed for total motility among the PC, FLAX, ASCA, and FLAX + ASCA treatment groups during the summer season. During the autumn season, the FLAX + ASCA group had a significantly (*p* < 0.05) higher (97.54 ± 1.77) total motility than that of the NC (86.63 ± 1.76) and the PC (91.05 ± 1.91) and AA (91.78 ± 1.82). Although total motility in FLAX + ASCA (97.15 ± 2.08) could not differ significantly (*p* > 0.05) from that of ASCA (94.30 ± 2.19), it was higher than that of the NC (90.15 ± 2.14).

Static spermatozoa were always higher (*p* < 0.05) in the NC during the spring (10.81 ± 2.18), summer (11.30 ± 1.84), autumn (13.11 ± 1.47), and winter (9.84 ± 1.79). The static motility of the NC in all four seasons did not match that of the PC. Whenever FLAX, ASCA, and FLAX + ASCA were introduced, a significant reduction was realized.

Sperm plasma membrane permeability and viability, as well as sperm abnormalities, were evaluated during four different seasons. Intact sperm cells were significantly (*p* < 0.05) higher during the winter (81.16 ± 1.18), autumn (80.18 ± 1.03), and summer (78.06 ± 1.24) seasons when compared to the spring (76.30 ± 1.42) season (Table 6). However, a non-significant (*p* > 0.05) difference was observed between the spring and summer seasons in terms of the intact sperm plasma membrane. The non-intact sperm plasma membrane was higher in the spring (23.70 ± 1.41), than in the autumn (19.82 ± 1.02) and winter (18.84 ± 1.17). No significant (*p* > 0.05) difference was observed for the non-intact sperm plasma membrane between the autumn (19.82 ± 1.02) and winter (18.84 ± 1.17) seasons. For sperm viability, live sperm cells were higher (*p* < 0.05) during the summer (85.04 ± 1.28) when compared to the spring (80.52 ± 1.28) and the autumn (80.94 ± 0.93) and winter (80.06 ± 1.07) seasons. The number of dead sperm cells was higher (*p* < 0.05) in the spring (18.99 ± 1.25) than in the autumn (18.93 ± 0.91) and winter (19.81 ± 1.04) seasons.

The effect of season and treatment diets on sperm plasma membrane permeability, viability, and sperm abnormality. Seasons had an effect on sperm plasma membrane permeability in the NC-treated group, with the winter (77.42 ± 2.36) outperforming the spring (70.97 ± 2.89), summer (74.80 ± 2.44), and autumn (71.51 ± 1.94) seasons (Table 7). The PC-treated group had winter (77.44 ± 2.50) and autumn (76.15 ± 2.11) outperform both the spring (68.24 ± 2.36) and the summer (62.68 ± 2.36) seasons for the intact sperm cells. The FLAX-treated group had a lower spring score (78.57 ± 2.79), and fewer intact sperm cells when compared to the summer (83.24 ± 2.32), autumn (84.59 ± 1.91), and winter (86.06 ± 2.26) seasons. In the ASCA-treated group, there was no significant difference (*p* > 0.05) between the spring (83.51 ± 2.94) and summer (83.01 ± 2.50), autumn (79.86 ± 2.01), and winter (78.19 ± 2.41) for intact sperm cells. The FLAX + ASCA had more intact sperm cells in the autumn (88.77 ± 1.96) than in the spring (80.24 ± 2.79) but did not differ from the summer (86.57 ± 2.32) or winter (86.67 ± 2.26) seasons.

The number of live sperm cells in the NC-fed groups was significantly higher (*p* < 0.05) in the summer season (74.80 ± 2.44), than in the spring (65.44 ± 2.61), and winter (65.33 ± 2.13), but not significantly different from those in the autumn season (71.50 ± 1.96). Summer (79.25 ± 2.13) and winter (72.92 ± 2.25) had higher live sperm cell counts than spring (71.97 ± 2.55) and autumn (70.79 ± 1.90) in the PC-fed group. The FLAX + ASCA treated groups had significantly higher live sperm cells during summer (97.00 ± 2.10), spring (91.27 ± 2.52), autumn (94.09 ± 1.77) and winter (91.19 ± 2.07) when compared to the counterpart treatment diets within a season. Nevertheless, live sperm cells in FLAX + ASCA could not differ among seasons (spring, summer, autumn, and winter).

The sperm abnormalities (primary, secondary, and tertiary) results can be found in Table 7. The NC-treated group had significantly higher primary sperm abnormalities (11.38 ± 0.76) when compared to the autumn (9.43 ± 0.62) season, but no significant difference (*p* > 0.05) when compared to the spring (11.33 ± 0.93) and summer (10.26 ± 0.78) seasons. The PC-treated group had significantly (*p* < 0.05) fewer primary sperm abnormalities during the summer (7.46 ± 0.76) than the autumn (10.11 ± 0.68) but could not differ from those of the spring (9.02 ± 0.91) and winter (9.02 ± 0.80). Both FLAX and FLAX + ASCA yielded a non-significant (*p* > 0.05) difference for the primary anomalies. In ASCA, spring (3.75 ± 0.94) and summer (4.04 ± 0.80) had significantly (*p* < 0.05) lower primary sperm abnormalities when compared to autumn (6.44 ± 0.64) and winter (5.96 ± 0.77).

The secondary sperm abnormalities in the NC-treated group could not differ significantly (*p* > 0.05) between the spring (11.42 ± 0.94), summer (10.14 ± 0.79), autumn (9.61 ± 0.63), and winter (11.49 ± 0.77) seasons. When looking at the PC, there were significant (*p* < 0.05) lower secondary sperm abnormalities during the summer (7.51 ± 0.77) compared to the winter (9.18 ± 0.81) and autumn (10.07 ± 0.69) seasons. The FLAX could not lead to any significant (*p* > 0.05) differences between seasons for the secondary sperm abnormalities. The dietary ASCA inclusion resulted in significantly lower secondary sperm abnormalities (*p* < 0.05) during spring (3.74 ± 0.96), summer (4.16 ± 081), and autumn (6.42 ± 0.65). Nevertheless, there was no significant (*p* > 0.05) difference between the autumn (6.42 ± 0.65) and winter (6.01 ± 0.79) seasons. The FLAX + ASCA could not lead to any significant differences (*p* > 0.05) among the seasons.

On the other hand, the tertiary sperm abnormalities in the NC group were significantly (*p* < 0.05) lower during the autumn (9.60 ± 0.63) when compared to the winter (11.56 ± 0.76) seasons. However, there was no significant (*p* > 0.05) difference between the spring (11.30 ± 0.93), summer (10.21 ± 0.79), and autumn (9.60 ± 0.63) seasons for tertiary sperm abnormalities. In the PC-treated group, there were significantly (*p* < 0.05) higher tertiary sperm abnormalities during the autumn (10.05 ± 0.68) and winter (9.15 ± 0.81) seasons when compared to the spring (9.14 ± 0.91) and summer (7.49 ± 0.76) seasons. The 5% flaxseed oil could not lead to any significant (*p* > 0.05) differences in all seasons (spring, summer, autumn, and winter). When looking at the ASCA, there were significant (*p* < 0.05) higher tertiary abnormalities during the autumn (6.49 ± 0.65) in comparison to the spring (3.75 ± 0.9) and summer (4.09 ± 0.81) seasons. There was no significant (*p* > 0.05) difference among seasons for tertiary sperm abnormalities.

The seasonal effect on the MDA level and testosterone level results are presented in Table 8. Seasonal effects on MDA levels were not significant (*p* > 0.05). Testosterone levels were significantly (*p* < 0.05) higher during the autumn (22.04 ± 1.94), when compared to the spring (16.41 ± 2.00) and summer (14.38 ± 2.04). However, there was no significant change (*p* > 0.05) in the testosterone levels during the autumn (22.04 ± 1.94) and winter (20.22 ± 2.00) seasons.

The effect of seasons and treatment diets on MDA and testosterone levels was evaluated. There was a higher MDA level in the NC (0.64 ± 0.10) during summer and winter (0.68 ± 0.10) as compared to the NC (0.33 ± 0.10) during the autumn season (Table 9). There was no significant difference (*p* > 0.05) observed among seasons for PC, FLAX, and ASCA. The FLAX + ASCA led to significant (*p* < 0.05) MDA levels during the spring season (0.27 ± 0.07) when compared to the winter season (0.54 ± 0.10). When the MDA levels were compared across the table (Table 9), there was a clear indication that the NC led to a significantly higher MDA level in all seasons except winter. The winter season had a non-significant (*p* > 0.05) MDA level between the NC (0.68 ± 0.10) and FLAX + ASCA (0.54 ± 0.10).

The testosterone levels in different seasons were followed by dietary supplementation with treatment diets (NC, PC, FLAX, ASCA, and FLAX + ASCA), and the results are available in Table 9. There was no significant difference in the testosterone levels when the NC and PC were supplemented. The FLAX during the autumn season had higher (29.02 ± 3.38) testosterone levels than other seasons on the same diet. The spring, summer, and winter seasons could not differ significantly (*p* > 0.05) from each other on FLAX. The summer (7.03 ± 3.74) and spring (12.44 ± 3.38) seasons in ASCA could not differ significantly (*p* > 0.05) and scored the lowest testosterone levels when compared to the autumn (23.37 ± 4.22) and winter (17.15 ± 3.38) seasons. The testosterone levels in FLAX + ASCA could not differ significantly (*p* > 0.05) with the seasons (spring, summer, autumn, and winter). However, testosterone levels during autumn (NC, 17.86 ± 3.38; PC, 22.64 ± 4.22; 5% flaxseed oil, 29.02 ± 3.19; ASCA, 23.37 ± 4.2; and FLAX + ASCA, 28.22 ± 3.19) outperformed all other seasons. On the other hand, the NC led to lower testosterone levels in all seasons when compared to the other diets (PC, FLAX, ASCA, and FLAX + ASCA).

The body weight and testicular dimensions were measured each season. There was no significant (*p* > 0.05) difference in body weight and scrotal circumference in different seasons. The scrotal length was significantly (*p* < 0.05) higher during the summer (13.50 ± 0.23), autumn (13.41 ± 0.13), and winter (13.69 ± 0.17) seasons, in comparison to that observed during the spring season (12.78 ± 0.23) (Table 10). The autumn season outperformed all other seasons for scrotal length (16.22 ± 0.13). However, no significant (*p* > 0.05) difference was observed between the spring (15.13 ± 0.23) and winter (15.68 ± 0.17) seasons. It was noteworthy that summer scored the lowest scrotal width (14.10 ± 0.23) of all the other seasons.

Body weights during the spring and summer seasons were not affected by the administered diets. However, the FLAX + ASCA resulted in a significantly (*p* < 0.05) higher body weight (64.78 ± 1.37), when compared to the ASCA (60.74 ± 1.37) and FLAX (58.40 ± 1.37) during the autumn season (Table 11). The FLAX + ASCA during the winter season resulted in a significantly (*p* < 0.05) higher body weight (66.78 ± 2.37) when compared to the ASCA (61.24 ± 1.50) and FLAX (58.06 ± 1.50). There was no influence of season on the body weight within the negative, PC, FLAX, and ASCA fed groups. Noteworthy, the 5% flaxseed oil + 4% ascorbic resulted in a significant (*p* < 0.05) higher body weight during the autumn (64.78 ± 1.37) and winter (66.78 ± 2.37) than that during the spring (58.45 ± 2.37) and summer (58.90 ± 2.37).

The scrotal circumference was measured for all treatment diet groups during all four seasons. The scrotal circumference during the spring season was significantly affected by the treatment diets, with the PC scoring the lowest (30.50 ± 0.62) than other dietary groups. The FLAX + ASCA (33.00 ± 0.62), ASCA (31.63 ± 0.62), and FLAX (32.50 ± 0.62) did not differ significantly (*p* > 0.05) amongst each other but were significantly (*p* < 0.05) higher than that of the negative (29.75 ± 0.62) and the PC (30.50 ± 0.62) during the spring season. The summer season resulted in a higher scrotal circumference when the FLAX + ASCA (32.88 ± 0.62) group was fed as compared to the ASCA (30.38 ± 0.62), PC (30.50 ± 0.62), and NC (29.44 ± 0.62) groups. The autumn season had a significantly higher (*p* < 0.05) scrotal circumference in the FLAX + ASCA (33.33 ± 0.36) and FLAX (32.75 ± 0.62) than that found in the negative (30.19 ± 0.36), PC (29.71 ± 0.36) and 4% ascorbic acid (30.96 ± 0.36). The winter season had a significantly higher (*p* < 0.05) scrotal circumference (33.63 ± 0.62) than other dietary groups. When the scrotal circumference was observed within treatment diets in different seasons, no significant difference (*p* > 0.05) was observed across all dietary groups.

The scrotal length differs significantly (*p* > 0.05) in all dietary treatments and seasons, except when FLAX + ASCA was used, where the spring season scored a significantly (*p* < 0.05) lower scrotal length (12.23 ± 0.51) than that of the summer (14.13 ± 0.51) and winter (13.75 ± 0.51) seasons. On the other hand, the scrotal width was affected by the seasons and the dietary treatments. The spring season resulted in a significantly lower (*p* < 0.05) scrotal width for the NC (14.50 ± 0.52) than other groups. There was no significant (*p* < 0.05) difference in all dietary groups during the summer. The FLAX + ASCA group had a significantly (*p* < 0.05) higher scrotal length during both the autumn (17.42 ± 0.30) and winter (17.38 ± 0.52) seasons. However, within the dietary groups, there was no significant (*p* > 0.05) effect of season.

## 4. Discussion

This is the first study to evaluate the effect of seasonal variations on semen quality, testosterone, and scrotal size following the administration of dietary ascorbic and omega-3 fatty acids to South African indigenous rams. The sperm volume could not differ with the seasons [21]. Nevertheless, the season has an impact on sheep reproductive efficiency, particularly in the temperate zone [39,40]. Small ruminants are greatly affected by environmental factors such as the amount of light or the photoperiod length. A decrease in the duration of natural light improves the concentration of melatonin during the autumn and winter months. This improves the regulation of the reproductive axis, increasing the responsiveness of the pineal gland and the secretion of melatonin. There are changes in the reproductive system of the male during the autumn and winter months, such as the production of viable spermatozoa, increased sexual behavior, resumption of testicular activities, and increased levels of reproductive hormones such as GnRH, LH, FSH, and testosterone [41]. However, in the current study, it was noteworthy that the semen volume observed was similar to that reported previously [21], but lower than that reported by Chella et al. [42]. Semen volume reached a peak of 1.20 ± 0.11 during the summer season when FLAX + ASCA combination was supplemented. These results differed from those reported by Belkadi et al. [43] and Zamiri et al. [44], where semen volumes peaked during the autumn season. The productivity of sheep is highly affected by the seasonality of their reproduction [23]. Chella et al. [42] reported that Zulu rams produce at least 0.7 ± 0.6 mL of semen volume during the non-breeding season and 1.1 ± 0.1 mL of semen volume during the breeding season, indicating a significant influence of season. It has been reported previously that the reduction in sexual behavior and spermatozoa quality takes place in sheep during the non-reproductive season [39].

Moreover, sexual behavior, testicular size, and semen quantity and quality are the main factors affecting the ram’s reproductive efficiency; the testicle size correlates with the semen volume [23]. On the other hand, Ngcobo et al. [21] observed that Zulu sheep produce 0.7 ± 0.5 mL of semen volume during the non-breeding season, primarily because rams are seasonal animals and reproductively active when daylight decreases due to the ratio of daylight to dark [45]. Similar results were observed by Samadian et al. [46], where supplementing long-chain polyunsaturated fatty acids improved sperm quality and quantity of fresh sperm and extended sperm quality after the breeding season through increasing docosahexaenoic acid (DHA) in rams’ sperm cells; however, DHA analysis on sperm cells was out of scope for this study. Furthermore, it increases rams’ seminal plasma antioxidant status and reduces sperm DNA fragmentation [47]. Therefore, based on these results, flaxseed oil can be classified as an antioxidant that can reverse the seasonal effect on semen volume.

Semen pH increased when treatment diets were introduced in all seasons. According to Al-Anazi et al. [48], semen pH varies with the season, being alkaline and significantly lower during autumn than in spring. However, ram semen pH should range between 5.9 and 7.3 when grazing on pastures [42]. According to Lu et al. [49], semen pH reflects the balance between the pH values of the different accessory gland secretions: alkaline seminal vesicular secretion and acidic prostate secretion. The semen pH observed in this study fell within normal limits in all seasons.

Sperm concentration in this study peaked during the spring season (1.28 ± 0.13) on the PC and was the lowest during the winter season (0.53 ± 0.11) on the NC. However, our results differed from those reported previously, where sperm concentration was higher during the autumn season [43]. Hedia et al. [50] found that sperm cell concentration reached its maximum quantity late in the winter. In South Africa, the winter season is considered a non-breeding season [21]. Sperm volume may influence sperm concertation. For example, when FLAX + ASCA combination was added to the current during spring, we saw a higher semen volume (1.20 ± 0.11) but a relatively low sperm concentration (0.88 ± 0.01). Flaxseed oil-enriched diets alter spermatogenesis even during the declining portion of the yearly reproductive season [27]. However, the low sperm concentration during spring is due to the seasonal variations in day length that affect testicular activity by influencing GnRH secretion and consequently gonadotropins [51].

In this study, progressive motility could not differ significantly throughout the year. Kulaksizi et al. [52] reported that progressive motility was higher during the breeding season than the non-breeding season. Sperm motility should be assessed as soon as possible after semen collection, preferably within 30 min, due to pH changes, dehydration effects, and temperature alteration effects on motility [53]. Progressive motility could be defined as sperm cells that are actively transiting either linearly or on a large scale, notwithstanding the speed [49]. Progressively motile sperm should be classified as rapid or slow, with >25 µm/s at 37 °C defining grade A spermatozoa [22]. The influence of season and treatment diets on progressive motility was evaluated. There was a significant difference in the NC, PC, and FLAX; however, ASCA and FLAX + ASCA led to non-significant differences. It is well known that the season has an effect on sperm quality [54], and flaxseed oil and natural antioxidants (vitamins C and E) are capable of improving the sperm quality of livestock [55]. In Chios rams, sperm motility could not differ between seasons [20], even after freezing and thawing [56]. Progressive motility can be associated with pregnancy [57]. According to Benmoula et al. [22], progressive motility is higher in the winter and summer. Moreover, other studies have reported lower progressive motility during the summer and spring [48]. Furthermore, in the same study, no significant differences were observed between winter and autumn for progressive motility; hence, it was concluded that Naimi and Najdi rams can be successfully bred during spring and autumn.

Progressive motility was influenced by different seasons; however, when treatment diets were introduced, there was a noticeable improvement across all seasons. Our results were in line with those previously reported, where progressive motility peaks during autumn and spring when rams are grazing on the natural veld or pasture [50,57,58]. Nevertheless, they differed from those reported by Benmoula et al. [22]. Notably, improvements in progressive motility were observed in this study because flaxseed oil contains linolenic acid, which is then converted to eicosapentaenoic acid (C20:5n-3) and docosahexaenoic acid (DHA). Therefore, problems associated with poor flock reproductive performance during certain seasons can be mimicked using exogenous flaxseed oil for lambing at any season of the year. Total sperm motility peaked in autumn on FLAX + ASCA (97.54 1.77) and winter on FLAX + ASCA (97.15 2.08), but it did not differ from spring and summer on the FLAX + ASCA group. Belkadi et al. [43] found high mass motility during the spring season.

Non-progressive motility could not differ with the season in the ASCA and FLAX + ASCA fed groups but was less than that of the negative control and the PC. The NPM could be described as sperm cells with no visible movements or those swimming in small circles [49]. On the other hand, total motility could not differ among seasons in the NC, PC, FLAX, and the FLAX + ASCA group. These results were in line with Benmoula et al.’s [22] report that season does not influence total motility in INRA180 rams. Moreover, provided that total motility is a sum of progressive motility and non-progressive motility [49], a similar trend in progressive motility and total motility was observed, as was expected. Since total motility is highly important for sperm cells, similar results were observed in static sperm cells [49]. Static sperm cells are those sperm cells that are not moving at all [49]. The seasons of spring and summer are naturally longer than those of autumn and winter [34]. However, there was a non-significant difference between spring, autumn, and winter in this study for static spermatozoa. This justifies the ability of flaxseed oil and ascorbic acid to improve semen quality throughout the year.

The morphology of sperm cells was first reported to affect fertility in livestock animals [59]. In the current study, sperm morphologies were classified as primary, secondary, and tertiary. Primary sperm abnormalities arise during spermatogenesis, secondary abnormalities after ejaculation, and tertiary abnormalities during in vitro sperm handling. Seasonal differences in sperm cell abnormalities could not exist. However, looking at the effect of treatment diets, primary, secondary, and tertiary abnormalities were higher in the NC group in comparison to other dietary groups, with FLAX + ASCA scoring the lowest regardless of the season. Poor semen quality can be a result of the testis producing abnormal spermatozoa, or it can be due to post-testicular damage to spermatozoa in the epididymis or the ejaculate from accessory gland secretions [60]. As a result, flaxseed oil in the form of long-chain polyunsaturated fatty acids validates its use to enhance sperm quality and abnormalities [31].

Malondialdehyde levels during the spring season were higher in the NC (0.53 ± 0.07) than in other treatment groups, with the lowest in FLAX + ASCA (0.27 ± 0.07) and FLAX (0.22 ± 0.07). Oxidative stress is a vital contributor to fertility problems in sperm cells via lipid peroxidation [61]. According to Colagar et al. [61], malondialdehyde is an indicator of lipid peroxidation, which may be a diagnostic tool for the analysis of infertility. Moreover, reactive oxygen species (ROS) as a result of lipid peroxidation alter the lipid concentration of the sperm plasma membrane and increase the detached acrosome [62]. The summer season had a significantly (*p* < 0.05) higher malondialdehyde level when the NC (0.65 ± 0.10) was fed. Malondialdehyde did not differ significantly (*p* > 0.05) between PC, FLAX, ASCA, and FLAX + ASCA during the summer season. Dietary influences on malondialdehyde levels were unclear during the autumn and winter seasons. There was no significant (*p* > 0.05) difference across all seasons as they interacted with breeds, with the exception of the difference between Zulu rams in spring (0.26 ± 0.06) and Zulu rams in autumn (0.44 ± 0.06).

Testosterone in this study was higher during the autumn and winter. Testosterone levels are known to peak during the breeding season [56]. Nevertheless, the polyunsaturated fatty acids (PUFAs) can mimic the testosterone levels across different seasons [63], and thus our results were expected because daylight length decreases during winter and autumn and activates melatonin functions, making these seasons known as the breeding season [64]. Testosterone is a male sexual hormone critical for spermatogenesis [65]. However, Belkhiri et al. [66] could not find any differences among seasons for testosterone levels. Mandiki et al. [67], on the other hand, reported low testosterone and follicle-stimulating hormone in Suffolk rams during the non-breeding season. Follicle-stimulating hormones control Sertoli cells to initiate proper spermatogenesis [68]. This hormone is produced by the pituitary gland and controlled by GnRH, which is regulated by the day length [45]. Gonadotropin concentration and circulation are influenced by the season [18], which undergoes a significant gonadal involution from the autumn to winter seasons [69]. The influence of treatment diets was measured, and it was observed that FLAX + ASCA led to higher testosterone levels than ASCA.

The assessment of breeding rams before breeding season helps to eliminate rams with hereditary defects and prevent mating with infertile rams [70]. This is necessary because male fertility is a critical contributor to flock breeding potential [71]. Numerous studies have reported seasonal variations in rams’ reproductive efficiency [43]. In the current study, body weight did not differ with seasons throughout the year. Similar results were observed in the Persian Karakul rams in different seasons of the year [72]. However, our results differed from those observed by Belkadi et al. [43], where rams during the spring season had a slightly higher body weight. These differences might be due to the fact that the rams used in the current study were mature rams raised at the research station. Bodyweight and the body condition score are critical in rams and should be evaluated 6–8 weeks before breeding season [70]. Furthermore, the time rams spend on sexual behavior to locate ewes on heat mating reduces the amount of time available for feeding [73], resulting in about 10–15% body weight loss during breeding season.

The scrotum circumference was also measured in the current study and was not affected by the seasons. Scrotum circumference and testicle size are vital during the breeding soundness evaluation of breeding rams [70]. This can be examined by physical palpation or measuring tape to evaluate the circumference of the scrotum. However, scrotal circumference has been different in different breeds and seasons. For instance, Aguirerre et al. [74] found that scrotal circumference is significantly larger during short days (30.90 ± 0.12) than that of longer days (29.59 ± 0.32) in Pelibuey rams. In Suffolk rams, scrotal circumference was bigger (31.54 ± 0.68) during the autumn season [69], whereas Karakul rams recorded 33.30 ± 1.4 scrotal circumference [75]. Therefore, our results suggest that dietary supplementation with flaxseed oil and ascorbic acid can reverse the effects of seasons on the scrotal circumference.

It also appears that nutrition plays a vital role in the reproductive processes and should be considered when raising animals or during the non-breeding season to prepare for the upcoming breeding season [17]. Vitamins, a vital class of nutrients, improve the reproductive performance of rams [70]. Male reproductive efficiency requires vitamin E to improve testicular tissues and spermatogenesis through its antioxidant properties and roles in prostaglandin synthesis and growth metabolism [76]. Flaxseed oil contains vitamin E as a natural antioxidant [24]. Moreover, supplementation with ascorbic acid as a natural antioxidant might explain the improvement seen in the FLAX and ASCA groups. Therefore, the poor results found in the negative diet might be due to a limited intake of balanced minerals, resulting in compromised reproductive performance. Rams have high levels of zinc, selenium, and cobalt for reproductive efficiency [76]. Flaxseed oil contains high levels of omega n-3 fatty acids, so supplementing with FLAX + ASCA was expected to improve scrotal circumference and semen quality even outside of breeding season months.

## 5. Conclusions

In conclusion, results from the current study demonstrated that seasons affect the semen quality, with the autumn and winter seasons producing superior semen quality in comparison to the spring and summer seasons. However, the addition of flaxseed oil and ascorbic acid seems to improve semen quality throughout the year, regardless of the season. The present study further demonstrated that the testosterone level and scrotal size are affected by the season, being actively produced during the winter and autumn seasons. Notably, supplementing flaxseed oil and ascorbic acid can be used to reverse the effects of seasons on semen quality.

## Figures and Tables

**Table 1:** Fatty acid profile of experimental diets.

Fatty Acid	Treatment Diets				
	NC (g/100 g)	PC (g/100 g)	FLAX (g/100 g)	ASCA (g/100 g)	FLAX + ASCA (g/100 g)
Lauric acid (12:0)	ND	0.53	ND	1.09	ND
Myristic acid (C14:0)	ND	0.36	ND	0.42	ND
Palmitic acid (C16:0)	9.70	12.9	6.52	11.60	6.80
Stearic acid (C18:0)	3.10	3.59	4.84	3.44	4.61
Cis Oleic acid (C18:1) n9	15.33	20.35	20.34	27.96	20.03
Cis Linoleic acid (C18:2) n6	19.40	44.0	16.68	37.4	19.21
Linoleic acid (C18:3) n6	ND	ND	<LOQ	ND	<LOQ
Linolenic acid (C18:3) n3	6.97	9.60	50.20	5.59	48.0
Arachidic acid (C20:0)	9.60	2.26	0.35	4.68	0.50
Eicosenoic acid (C20:1) n9	ND	0.52	<LOQ	0.58	<LOQ
Eicosadienoic acid (C20:1) n6	ND	ND	ND	<LOQ	ND
Arachidonic acid (C20:4) n6	0.33	<LOQ	ND	<LOQ	ND
Eicosapentaenoic acid (C20:5) n3	0.53	0.33	ND	0.39	ND
Heneicosanoic acid (C21:0)	ND	<LOQ	ND	0.32	ND
Behenic acid (C22:0)	2.24	0.89	<LOQ	0.90	<LOQ
Erucic acid (C22:1) n9	ND	ND	ND	0.37	ND
Docosahexaenoic acid (C22:6) n3	ND	ND	ND	ND	ND
Lignoceric acid (C24:0)	1.68	0.66	<LOQ	0.69	<LOQ
Cerotic acid (C26:0)	1.59	0.52	-	-	-
Octacosanoic acid (C28:0)	2.58	0.60	-	-	-

NB: Values below the limit of quantitation cannot be accurately quantified. ND–Nondetectable, LOQ–Limit of Quantification = 0.28 g fatty acids/100 g fatty acids. NC–Negative control, PC–Positive control, FLAX–5% Flaxseed oil, ASCA–4% Ascorbic acid, FLAX + ASCA–5% Flaxseed oil + 4% Ascorbic acid.

**Table 2:** The effect of season on semen volume, pH, and sperm concentration.

Semen Parameters	Seasons of the Year				
	Spring	Summer	Autumn	Winter	*p*-Value
Semen Volume (mL)	0.88 ± 0.06	0.91 ± 0.06	0.92 ± 0.05	0.92 ± 0.05	>0.05
Semen pH	6.44 ± 0.11 ^a^	6.32 ± 0.10 ^ab^	6.07 ± 0.08 ^b^	6.10 ± 0.09 ^b^	<0.05
Sperm Concentration (×10^9^)	0.93 ± 0.06 ^a^	0.84 ± 0.06 ^ab^	0.71 ± 0.05 ^b^	0.70 ± 0.05 ^b^	<0.05

NB–^ab^ Means with different superscripts within the row differ significantly (*p* < 0.05).

**Table 3:** The effect of season and treatment diets on the semen volume, pH, and sperm concentration.

Semen Parameters	Season	NC	PC	FLAX	ASCA	FLAX + ASCA	*p*-Value
Semen volume (mL)	Spring	0.55 ± 0.11 ^i^	0.72 ± 0.11 ^c–h^	1.03 ± 0.13 ^a–g^	0.96 ± 0.13 ^a–g^	1.20 ± 0.11 ^ab^	<0.05
	Summer	0.55 ± 0.11 ^hi^	0.72 ± 0.11 ^f–h^	1.10 ± 0.11 ^a–c^	0.97 ± 0.11 ^a–g^	1.20 ± 0.11 ^a^	<0.05
	Autumn	0.79 ± 0.09 ^d–h^	0.74 ± 0.09 ^fh^	0.93 ± 0.09 ^a–g^	1.01 ± 0.09 ^a–e^	1.12 ± 0.09 ^a–c^	<0.05
	Winter	0.88 ± 0.11 ^b–g^	0.75 ± 0.11 ^e–h^	0.90 ± 1.10 ^b–g^	1.04 ± 0.11 ^a–e^	1.03 ± 0.10 ^a–d^	<0.05
*p*-value		<0.05	>0.05	>0.05	>0.05	>0.05	
Semen pH	Spring	5.85 ± 0.23 ^f^	6.17 ± 0.22 ^b–f^	6.64 ± 0.22 ^ab^	7.00 ± 0.23 ^ab^	6.55 ± 0.18 ^a–d^	<0.05
	Summer	6.25 ± 0.19 ^b–f^	6.21 ± 0.19 ^b–f^	6.16 ± 0.18 ^b–f^	6.44 ± 0.20 ^b–e^	6.54 ± 0.18 ^a–c^	<0.05
	Autumn	6.17 ± 0.15 ^b–f^	6.00 ± 0.17 ^d–f^	5.99 ± 0.15 ^ef^	6.11 ± 0.16 ^c–f^	6.08 ± 0.16 ^c–f^	<0.05
	Winter	6.02 ± 0.19 ^c–f^	5.99 ± 0.20 ^d–f^	6.08 ± 0.18 ^b–f^	6.17 ± 0.19 ^b–f^	6.22 ± 0.18 ^b–f^	>0.05
*p*-value		>0.05	>0.05	>0.05	<0.05	<0.05	
Sperm conc. (×10^9^)	Spring	0.69 ± 0.13 ^b–e^	1.28 ± 0.13 ^a^	0.87 ± 0.12 ^b–d^	0.75 ± 0.09 ^ab^	0.81 ± 0.10 ^b–e^	<0.05
	Summer	0.94 ± 0.11 ^bc^	0.69 ± 0.11 ^c–e^	0.72 ± 0.10 ^b–e^	0.96 ± 0.11 ^bc^	0.88 ± 0.10 ^b–d^	>0.05
	Autumn	0.65 ± 0.09 ^de^	0.69 ± 0.09 ^b–e^	0.72 ± 0.09 ^b–e^	0.75 ± 0.10 ^b–e^	0.76 ± 0.09 ^b–e^	>0.05
	Winter	0.53 ± 0.11 ^e^	0.76 ± 0.11 ^b–e^	0.81 ± 1.01 ^b–e^	0.71 ± 0.11 ^b–e^	0.68 ± 0.10 ^b–e^	>0.05
*p*-value		<0.05	<0.05	>0.05	>0.05	>0.05	

NB–^abcdefghi^ Means with different superscripts across the table within the same cell differ significantly (*p* < 0.05). NC–Negative control, PC–Positive control, FLAX–5% Flaxseed oil, ASCA–4% Ascorbic acid, FLAX + ASCA–5% Flaxseed oil + 4% Ascorbic acid.

**Table 4:** The effect of season on sperm motility parameters.

Sperm Parameters	Seasons of the Year				*p*-Value
	Spring	Summer	Autumn	Winter	
Sperm Progression (%)					
PM	60.52 ± 2.62	62.78 ± 2.28	66.88 ± 1.91	64.02 ± 2.18	>0.05
NPM	30.48 ± 2.22 ^ab^	31.47 ± 1.94 ^a^	25.28 ± 1.62 ^b^	28.79 ± 1.85 ^ab^	<0.05
TM	91.01 ± 1.29 ^b^	94.25 ± 1.12 ^a^	92.16 ± 0.94 ^ab^	92.81 ± 1.07 ^ab^	<0.05
Static	8.68 ± 1.07 ^a^	5.48 ± 0.94 ^b^	7.89 ± 0.78 ^ab^	6.42 ± 0.90 ^ab^	<0.05

NB–^ab^ Means with different superscripts within the row differ significantly (*p* < 0.5). PM–progressive motility, NPM–non-progressive motility, TM–total motility.

**Table 5:** The effect of season and treatment diets on sperm motility parameters.

Semen Parameters	Season	NC	PC	FLAX	ASCA	FLAX + ASCA	*p*-Value
Sperm Progression (%)							
PM	Spring	42.84 ± 5.32 ^e^	62.17 ± 5.21 ^a–d^	63.00 ± 5.14 ^a–c^	64.38 ± 5.41 ^a–c^	70.23 ± 5.14 ^ab^	<0.05
	Summer	49.38 ± 4.49 ^de^	60.15 ± 4.36 ^b–d^	62.24 ± 4.28 ^a–c^	73.66 ± 4.60 ^a^	68.49 ± 4.28 ^ab^	<0.05
	Autumn	63.26 ± 3.58 ^a–c^	63.30 ± 3.88 ^a–c^	67.38 ± 3.53 ^ab^	69.08 ± 3.70 ^ab^	71.37 ± 3.61 ^ab^	>0.05
	Winter	62.46 ± 4.35 ^a–c^	63.17 ± 4.60 ^a–c^	53.83 ± 4.16 ^c–e^	71.13 ± 4.45 ^ab^	69.50 ± 4.23 ^ab^	<0.05
*p*-value		<0.05	<0.05	<0.05	>0.05	>0.05	
NPM	Spring	46.09 ± 4.52 ^a^	29.40 ± 4.42 ^b–f^	28.99 ± 4.36 ^b–f^	23.42 ± 4.60 ^d–f^	24.53 ± 4.36 ^d–f^	<0.05
	Summer	39.05 ± 3.81 ^ab^	32.96 ± 3.70 ^b–e^	33.31 ± 3.63 ^b–d^	23.53 ± 3.91 ^ef^	28.49 ± 3.63 ^c–f^	<0.05
	Autumn	23.37 ± 3.04 ^ef^	27.75 ± 3.30 ^d–f^	26.42 ± 2.99 ^d–f^	22.70 ± 3.14 ^f^	26.17 ± 3.06 ^d–f^	>0.05
	Winter	27.69 ± 3.69 ^c–f^	28.55 ± 3.91 ^b–f^	36.88 ± 3.53 ^a–c^	23.17 ± 3.78 ^ef^	27.66 ± 3.59 ^d–f^	<0.05
*p*-value		<0.05	<0.05	<0.05	>0.05	>0.05	
TM	Spring	88.93 ± 2.62 ^e–h^	91.56 ± 2.56 ^a–h^	91.99 ± 2.53 ^a–h^	87.79 ± 2.66 ^gh^	94.76 ± 2.53 ^a–f^	<0.05
	Summer	88.43 ± 2.21 ^f–h^	93.11 ± 2.14 ^a–g^	95.55 ± 2.10 ^a–e^	97.20 ± 2.26 ^a–c^	96.98 ± 2.10 ^a–d^	<0.05
	Autumn	86.63 ± 1.76 ^h^	91.05 ± 1.91 ^c–h^	93.80 ± 1.73 ^a–g^	91.78 ± 1.82 ^d–g^	97.54 ± 1.77 ^a^	<0.05
	Winter	90.15 ± 2.14 ^e–h^	91.72 ± 2.26 ^b–h^	90.71 ± 2.05 ^e–h^	94.30 ± 2.19 ^a–g^	97.15 ± 2.08 ^a–d^	<0.05
*p*-value		>0.05	>0.05	>0.05	<0.05	>0.05	
Static	Spring	10.81 ± 2.18 ^a–d^	7.99 ± 2.14 ^a–f^	7.72 ± 2.11 ^b–g^	11.93 ± 2.22 ^ab^	4.95 ± 2.11 ^d–g^	<0.05
	Summer	11.30 ± 1.84 ^a–c^	6.62 ± 1.79 ^c–g^	4.15 ± 1.76 ^e–g^	2.60 ± 1.89 ^g^	2.72 ± 1.76 ^fg^	<0.05
	Autumn	13.11 ± 1.47 ^a^	9.22 ± 1.59 ^a–d^	6.35 ± 1.45 ^c–g^	7.92 ± 1.52 ^b–e^	2.84 ± 1.48 ^fg^	<0.05
	Winter	9.84 ± 1.79 ^a–d^	8.55 ± 1.89 ^a–e^	5.44 ± 1.71 ^d–g^	5.40 ± 1.83 ^d–g^	2.93 ± 1.74 ^fg^	<0.05
*p*-value		<0.05	<0.05	<0.05	<0.05	<0.05	

NB–^abcdefgh^ Means with different superscripts across the table within the same cell differ significantly (*p* < 0.05). PM–progressive motility, NPM–non-progressive motility, TM–total motility. NC–Negative control, PC–Positive control, FLAX–5% Flaxseed oil, ASCA–4% Ascorbic acid, FLAX + ASCA–5% Flaxseed oil + 4% Ascorbic acid.

**Table 6:** The effect of season on sperm cell plasma membrane integrity, viability, and morphology.

Semen Parameters	Seasons of the Year				*p*-Value
	Spring	Summer	Autumn	Winter	
Sperm Plasma Membrane (%)					
Intact	76.30 ± 1.42 ^b^	78.06 ± 1.24 ^ab^	80.18 ± 1.03 ^a^	81.16 ± 1.18 ^a^	<0.05
Sperm viability (%)					
Live	80.52 ± 1.28 ^b^	85.04 ± 1.12 ^a^	80.94 ± 0.93 ^b^	80.06 ± 1.07 ^b^	<0.05
Sperm Abnormalities (%)					
Primary	6.29 ± 0.46	5.84 ± 0.40	6.74 ± 0.33	6.69 ± 0.38	>0.05
Secondary	6.30 ± 0.46	5.87 ± 0.40	6.78 ± 0.34	6.78 ± 0.39	>0.05
Tertiary	6.31 ± 0.46	5.86 ± 0.40	6.78 ± 0.33	6.75 ± 0.38	>0.05

NB–^ab^ Means with different superscripts within the row differ significantly (*p* < 0.05).

**Table 7:** The effect of season and treatment diets on sperm cell plasma membrane integrity, viability, and morphology.

Semen Parameters	Season	NC	PC	FLAX	ASCA	FLAX + ASCA	*p*-Value
Sperm plasma membrane permeability (%)							
Intact	Spring	70.97 ± 2.89 ^i^	68.24 ± 2.82 ^ij^	78.57 ± 2.79 ^c–g^	83.51 ± 2.94 ^a–e^	80.24 ± 2.79 ^b–f^	<0.05
	Summer	74.80 ± 2.44 ^f–i^	62.68 ± 2.36 ^j^	83.24 ± 2.32 ^a–d^	83.01 ± 2.50 ^a–d^	86.57 ± 2.32 ^ab^	<0.05
	Autumn	71.51 ± 1.94 ^hi^	76.15 ± 2.11 ^e–h^	84.59 ± 1.91 ^a–c^	79.86 ± 2.01 ^c–f^	88.77 ± 1.96 ^a^	<0.05
	Winter	77.42 ± 2.36 ^d–g^	77.44 ± 2.50 ^d–h^	86.06 ± 2.26 ^ab^	78.19 ± 2.41 ^d–g^	86.67 ± 2.26 ^ab^	<0.05
*p*-value		<0.05	<0.05	<0.05	>0.05	<0.05	
Sperm viability (%)							
Live	Spring	65.44 ± 2.61 ^ij^	71.97 ± 2.55 ^h–j^	86.11 ± 2.52 ^c–e^	87.83 ± 2.65 ^b–d^	91.27 ± 2.52 ^a–c^	<0.05
	Summer	72.27 ± 2.20 ^h^	79.25 ± 2.13 ^fg^	87.88 ± 2.10 ^cd^	88.78 ± 2.25 ^bc^	97.00 ± 2.10 ^a^	<0.05
	Autumn	71.50 ± 1.76 ^hi^	70.79 ± 1.90 ^hij^	86.90 ± 1.73 ^cd^	81.41 ± 1.81 ^ef^	94.09 ± 1.77 ^ab^	<0.05
	Winter	65.33 ± 2.13 ^j^	72.92 ± 2.25 ^gh^	88.20 ± 2.04 ^cd^	82.66 ± 2.18 ^d–f^	91.19 ± 2.07 ^bc^	<0.05
*p*-value		>0.05	>0.05	>0.05	<0.05	<0.05	
Sperm abnormalities (%)							
Primary	Spring	11.33 ± 0.93 ^ab^	9.02 ± 0.91 ^a–c^	4.31 ± 0.90 ^f–h^	3.75 ± 0.94 ^f–h^	3.02 ± 0.90 ^gh^	<0.05
	Summer	10.26 ± 0.78 ^ab^	7.46 ± 0.76 ^cd^	4.66 ± 0.75 ^e–h^	4.04 ± 0.80 ^f–h^	2.81 ± 0.76 ^gh^	<0.05
	Autumn	9.43 ± 0.62 ^bc^	10.11 ± 0.68 ^ab^	4.65 ± 0.61 ^fh^	6.44 ± 0.64 ^de^	3.07 ± 0.63 ^gh^	<0.05
	Winter	11.38 ± 0.76 ^a^	9.02 ± 0.80 ^bc^	4.05 ± 0.72 ^f–h^	5.96 ± 0.77 ^d–f^	3.03 ± 0.74 ^gh^	<0.05
*p*-value		<0.05	<0.05	>0.05	<0.05	>0.05	
Secondary	Spring	11.42 ± 0.94 ^ab^	9.09 ± 0.77 ^a–c^	4.20 ± 0.91 ^f–h^	3.74 ± 0.96 ^f–h^	3.07 ± 0.91 ^gh^	<0.05
	Summer	10.14 ± 0.79 ^ab^	7.51 ± 0.77 ^cd^	4.70 ± 0.76 ^e–h^	4.16 ± 0.81 ^f–h^	2.84 ± 0.76 ^gh^	<0.05
	Autumn	9.61 ± 0.63 ^ab^	10.07 ± 0.69 ^ab^	4.71 ± 0.62 ^e–g^	6.42 ± 0.65 ^de^	3.10 ± 0.64 ^h^	<0.05
	Winter	11.49 ± 0.77 ^a^	9.18 ± 0.81 ^bc^	4.14 ± 0.74 ^f–h^	6.01 ± 0.79 ^d–f^	3.11 ± 0.75 ^gh^	<0.05
*p*-value		>0.05	<0.05	>0.05	<0.05	>0.05	
Tertiary	Spring	11.30 ± 0.93 ^ab^	9.14 ± 0.91 ^bc^	4.31 ± 0.90 ^f–h^	3.75 ± 0.95 ^f–h^	3.06 ± 0.90 ^gh^	<0.05
	Summer	10.21 ± 0.79 ^ab^	7.49 ± 0.76 ^cd^	4.68 ± 0.75 ^e–h^	4.09 ± 0.81 ^f–h^	2.84 ± 0.75 ^gh^	<0.05
	Autumn	9.60 ± 0.63 ^b^	10.05 ± 0.68 ^ab^	4.71 ± 0.62 ^fg^	6.49 ± 0.65 ^de^	3.06 ± 0.63 ^h^	<0.05
	Winter	11.56 ± 0.76 ^a^	9.15 ± 0.81 ^bc^	4.09 ± 0.73 ^f–h^	5.99 ± 0.78 ^d–f^	2.99 ± 0.74 ^gh^	<0.05
*p*-value		<0.05	<0.05	>0.05	<0.05	>0.05	

NB–^abcdefghij^ Means with different superscripts across the table within the same cell differ significantly (*p* < 0.05). NC–Negative control, PC–Positive control, FLAX–5% Flaxseed oil, ASCA–4% Ascorbic acid, FLAX + ASCA–5% Flaxseed oil + 4% Ascorbic acid.

**Table 8:** The effect of season on malondialdehyde and testosterone levels.

Parameters	Spring	Summer	Autumn	Winter	*p*-Value
Malondialdehyde (nmol/mL)	0.31 ± 0.03	0.42 ± 0.05	0.37 ± 0.04	0.42 ± 0.05	>0.05
Testosterone levels (ng/mL)	16.41 ± 2.00 ^bc^	14.38 ± 2.04 ^c^	22.04 ± 1.94 ^a^	20.22 ± 2.00 ^ab^	<0.05

NB–^abc^ Means with different superscripts within the row differ significantly (*p* < 0.05).

**Table 9:** The effect of season and treatment diets on malondialdehyde and testosterone level.

Parameters	Seasons	NC	PC	FLAX	ASCA	FLAX + ASCA	*p*-Value
Malondialdehyde (nmol/mL)	Spring	0.53 ± 0.07 ^a–c^	0.16 ± 0.07 ^g^	0.22 ± 0.68 ^e–g^	0.37 ± 0.07 ^c–f^	0.27 ± 0.07 ^e–g^	<0.05
	Summer	0.64 ± 0.10 ^a^	0.37 ± 0.10 ^b–g^	0.38 ± 0.10 ^b–g^	0.36 ± 0.10 ^b–g^	0.33 ± 0.10 ^c–g^	<0.05
	Autumn	0.33 ± 0.10 ^c–g^	0.29 ± 0.07 ^d–g^	0.19 ± 0.07 ^fg^	0.62 ± 0.10 ^a–c^	0.41 ± 0.07 ^b–e^	<0.05
	Winter	0.68 ± 0.10 ^a^	0.35 ± 0.10 ^c–g^	0.11 ± 0.10 ^g^	0.42 ± 0.10 ^a–f^	0.54 ± 0.10 ^a–d^	<0.05
*p*-value		<0.05	>0.05	>0.05	<0.05	<0.05	
Testosterone levels (ng/mL)	Spring	11.84 ± 3.38 ^c–e^	14.44 ± 3.19 ^b–e^	17.79 ± 3.38 ^b–d^	12.44 ± 3.38 ^cde^	28.31 ± 3.38 ^a^	<0.05
	Summer	9.92 ± 3.38 ^de^	13.55 ± 3.38 ^b–e^	15.00 ± 3.38 ^b–e^	7.03 ± 3.74 ^e^	22.48 ± 3.38 ^ab^	<0.05
	Autumn	17.86 ± 3.38 ^b–d^	22.64 ± 3.38 ^ab^	29.02 ± 3.38 ^a^	23.37 ± 4.22 ^ab^	28.22 ± 3.19 ^a^	<0.05
	Winter	8.94 ± 3.38 ^de^	19.66 ± 3.38 ^a–c^	14.11 ± 3.38 ^b–e^	17.15 ± 3.38 ^b–d^	23.01 ± 3.74 ^ab^	<0.05
*p*-value		>0.05	>0.05	<0.05	<0.05	>0.05	

NB–^abcdefg^ Means with different superscripts across the table within the same cell differ significantly (*p* < 0.05). NC–Negative control, PC–Positive control, FLAX–5% Flaxseed oil, ASCA–4% Ascorbic acid, FLAX + ASCA–5% Flaxseed oil + 4% Ascorbic acid.

**Table 10:** The effect of season on body weight and scrotal size.

Parameters	Spring	Summer	Autumn	Winter	*p*-Value
Body weight (kg)	60.21 ± 1.06	60.70 ± 1.06	62.14 ± 0.61	62.21 ± 0.78	>0.05
Scrotal circumference (cm)	31.45 ± 0.28	30.97 ± 0.28	31.16 ± 0.16	31.12 ± 0.20	>0.05
Scrotal length (cm)	12.78 ± 0.23 ^b^	13.50 ± 0.23 ^a^	13.41 ± 0.13 ^a^	13.69 ± 0.17 ^a^	<0.05
Scrotal width (cm)	15.13 ± 0.23 ^b^	14.10 ± 0.23 ^c^	16.22 ± 0.13 ^a^	15.68 ± 0.17 ^b^	<0.05

NB–^abc^ Means with different superscripts within the row differ significantly (*p* < 0.05).

**Table 11:** The effect of season and treatment diets on body weight and scrotal size.

Parameters	Seasons	NC	PC	FLAX	ASCA	FLAX + ASCA	*p*-Value
Body weight (kg)	Spring	60.48 ± 2.37 ^a–g^	63.68 ± 2.37 ^a–f^	57.93 ± 2.37 ^e–g^	60.50 ± 2.37 ^a–g^	58.45 ± 2.37 ^de^	>0.05
	Summer	62.18 ± 2.37 ^a–g^	65.05 ± 2.37 ^a–d^	60.00 ± 2.37 ^b–g^	57.38 ± 2.37 ^e–g^	58.90 ± 2.37 ^c–g^	>0.05
	Autumn	62.77 ± 1.37 ^a–f^	64.06 ± 1.37 ^a–c^	58.40 ± 1.37 ^e–g^	60.74 ± 1.37 ^c–g^	64.78 ± 1.37 ^ab^	<0.05
	Winter	63.01 ± 1.63 ^a–f^	61.98 ± 1.50 ^a–g^	58.06 ± 1.50 ^g^	61.24 ± 1.50 ^b–g^	66.78 ± 2.37 ^a^	<0.05
*p*-value		>0.05	>0.05	>0.05	>0.05	<0.05	
Scrotal circumference (cm)	Spring	29.75 ± 0.62 ^f–h^	30.50 ± 0.62 ^d–h^	32.50 ± 0.62 ^a–c^	31.63 ± 0.62 ^b–e^	33.00 ± 0.62 ^ab^	<0.05
	Summer	29.44 ± 0.62 ^gh^	29.44 ± 0.62 ^gh^	32.75 ± 0.62 ^a–c^	30.38 ± 0.62 ^d–h^	32.88 ± 0.62 ^ab^	<0.05
	Autumn	30.19 ± 0.36 ^f–h^	29.71 ± 0.36 ^gh^	31.63 ± 0.36 ^b–d^	30.96 ± 0.36 ^d–f^	33.33 ± 0.36 ^a^	<0.05
	Winter	30.35 ± 0.42 ^e–g^	29.20 ± 0.39 ^h^	31.40 ± 0.39 ^c–e^	31.03 ± 0.39 ^d–f^	33.63 ±0.62 ^a^	<0.05
*p*-value		>0.05	>0.05	>0.05	>0.05	<0.05	
Scrotal length (cm)	Spring	13.13 ± 0.51 ^a–c^	12.63 ± 0.51 ^bc^	12.88 ± 0.51 ^a–c^	13.13 ± 0.51 ^a–c^	12.23 ± 0.51 ^c^	>0.05
	Summer	13.50 ± 0.51 ^a–c^	13.13 ± 0.51 ^a–c^	14.00 ± 0.51 ^ab^	12.75 ± 0.51 ^a–c^	14.13 ± 0.51 ^a^	>0.05
	Autumn	13.44 ± 0.30 ^ab^	13.29 ± 0.30 ^a–c^	13.63 ± 0.30 ^ab^	13.48 ± 0.30 ^ab^	13.23 ± 0.30 ^a–c^	>0.05
	Winter	13.41 ± 0.35 ^ab^	13.45 ± 0.33 ^ab^	13.90 ± 0.33 ^a^	13.93 ± 0.33 ^a^	13.75 ± 0.51 ^ab^	>0.05
*p*-value		>0.05	>0.05	>0.05	>0.05	<0.05	
Scrotal width (cm)	Spring	14.50 ± 0.52 ^f–h^	15.13 ± 0.52 ^d–g^	16.38 ± 0.52 ^a–e^	15.13 ± 0.52 ^d–g^	15.44 ± 0.52 ^c–g^	<0.05
	Summer	13.38 ± 0.52 ^h^	13.38 ± 0.52 ^h^	14.31 ± 0.52 ^gh^	14.81 ± 0.52 ^f–h^	14.50 ± 0.52 ^f–h^	>0.05
	Autumn	15.46 ± 0.30 ^d–g^	15.50 ± 0.30 ^d–f^	16.46 ± 0.30 ^bc^	16.25 ± 0.30 ^b–d^	17.42 ± 0.30 ^a^	<0.05
	Winter	15.53 ± 0.36 ^d–g^	14.70 ± 0.33 ^fg^	15.50 ± 0.33 ^d–g^	15.30 ± 0.33 ^e–g^	17.38 ± 0.52 ^ab^	<0.05
*p*-value		<0.05	<0.05	<0.05	<0.05	<0.05	

NB–^abcdefgh^ Means with different superscripts across the table in the same cell differ significantly (*p* < 0.05). NC–Negative control, PC–Positive control, FLAX–5% Flaxseed oil, ASCA–4% Ascorbic acid, FLAX + ASCA–5% Flaxseed oil + 4% Ascorbic acid.

## Data Availability

The Tshwane University of Technology (TUT) and the Agricultural Research Council, Irene (ARC) remain the owners of any intellectual property as a result of this study. No information is allowed to be used without the prior consent of TUT, ARC, and DALRRD.
